# Multifactorial, Site-Specific Recurrence Models after Radical Cystectomy for Urothelial Carcinoma: External Validation in a Cohort of Korean Patients

**DOI:** 10.1371/journal.pone.0100491

**Published:** 2014-06-17

**Authors:** Hyung Suk Kim, Myong Kim, Chang Wook Jeong, Cheol Kwak, Hyeon Hoe Kim, Ja Hyeon Ku

**Affiliations:** Department of Urology, Seoul National University College of Medicine, Seoul, Korea; Van Andel Institute, United States of America

## Abstract

**Purpose:**

The aim of this study was to evaluate the accuracy of site-specific recurrence models after radical cystectomy in the Korean population.

**Materials and Methods:**

We conducted a review of an electronic medical record of 572 patients who underwent radical cystectomy for urothelial carcinoma of the bladder. Primary end point was the site-specific recurrence after radical cystectomy.

**Results:**

The median follow-up in the validation cohort was 42.3 months (interquartile range: 23.0–89.3 months). During the follow-up period, there were 165 patients (28.8%), 85 (14.9%), 31 (5.4%), and 78 (13.6%) who recurred in abdomen/pelvis, thoracic region, upper urinary tract, and bone, respectively. The c-indices of abdomen/pelvis, thoracic region, upper urinary tract, and bone models 3 years after radical cystectomy were 0.69 (95% confidence interval [CI], 0.65–0.73), 0.69 (95% CI, 0.64–0.75), 0.61 (95% CI, 0.52–0.69), and 0.65 (95% CI, 0.59–0.71), respectively. Kaplan-Meier curves demonstrated that models discriminated well and log-rank test were all highly significant (all p<0.001), except upper urinary tract model (p = 0.366). Decision curve analysis revealed that the use of prediction models for abdomen/pelvis, thoracic region, and bone recurrence was associated with net benefit gains relative to the treat-all strategy, but not the model for upper urinary tract recurrence.

**Conclusions:**

Abdomen/pelvis, thoracic region, and bone models demonstrate moderate discrimination, adequate calibration, and meaningful net benefit gains, whereas upper urinary tract model does not seem applicable to patients from Asia because it has suboptimal accuracy.

## Introduction

It is estimated that 72,570 new cases of bladder cancer will be diagnosed and 15,210 patients will die of their disease in the United States in 2013 [1]. In Korea, 3,415 new cases of bladder cancer, which consist of 2,752 males and 663 females, was diagnosed and 1,100 bladder cancer related deaths occurred during 2010 [2]. At the time of diagnosis, 25–30% of bladder tumors are found to be muscle-invasive [3]. Radical cystectomy is the standard care treatment for patients with muscle-invasive and some patients with high-risk non-muscle invasive bladder cancer. Nevertheless, up to 50% of patients experience disease recurrence after radical cystectomy [4], [5]. The aggressive natural behavior of disease recurrences after radical cystectomy results in poor prognosis [6]. Characterizing recurrence patterns after radical cystectomy is critical for patient counseling and developing evidence-based surveillance guidelines [7].

For tailoring patient-specific disease surveillance, scoring algorithms of site-specific disease recurrence after radical cystectomy have been designed [8]. Sites of disease recurrence in the study were classified into four locations; abdomen/pelvis, thoracic region, upper urinary tract, and bone. To our knowledge, no validation of models has been published to date in the literature to improve the decision-making ability of clinicians caring for patients. Furthermore, since the research of developing the models were entirely based on American population, the generalization of the models to external cohorts of patients with different characteristics is questionable.

The aim of this study was to evaluate the accuracy of site-specific recurrence models after radical cystectomy in the Korean population and to explore the applicability of the models in different clinical environment.

## Materials and Methods

### Ethics Statement

This study design and the use of patients' information stored in the hospital database were approved by the Institutional Review Board (IRB) at the Seoul National University Hospital. The approval number is H-1403-032-563. We were given exemption from getting informed consents by the IRB because the present study is a retrospective study and personal identifiers were completely removed and the data were analyzed anonymously. Our study was conducted according to the ethical standards laid down in the 1964 Declaration of Helsinki and its later amendments.

### Study cohort

We conducted a review of an electronic medical record of all 622 patients who underwent radical cystectomy for bladder cancer at Seoul National University Hospital from January 2001 through December 2001. Our methods for surgery, pathology review, and follow-up have previously been described in detail [9]. Our exclusion criteria were non-urothelial carcinoma, presentation as distant metastasis, and no documentation of all variables as required by each model and/or unavailable pathology. Patients with detectable disease within 30 days of cystectomy were also excluded from the study because this was likely present at the time of cystectomy [8]. Therefore, the validation cohort comprised 572 patients. The demographic data for model development cohort in comparison to external validation cohort is shown in Table 1. Pathologic stage was determined according to the 2009 American Joint Committee on Cancer (AJCC) staging system. Therefore, we re-reviewed the pathologic stage of patients with pT4 in the 2002 AJCC staging system. Consistent with the 2009 AJCC staging system, tumors invading directly into the prostate from the bladder were defined as pT4a, whereas prostate urethra, ducts, and/or stromal involvement with urothelial carcinoma independent of the primary bladder tumor were evaluated separately [8].

**Table 1:** Patient characteristics.

Characteristics	Development cohort	Validation cohort
Total	1388 (100)	572 (100)
Macroscopic hematuria	856 (62)	471 (82)
Occupational radiation exposure	26 (2)	0 (0)
Sex		
Men	1117 (80)	502 (88)
Women	271 (20)	70 (12)
Body mass index		
<20	39 (3)	74 (13)
20–25	418 (30)	335 (59)
25–30	644 (46)	147 (26)
30–35	225 (16)	9 (2)
>35	62 (5)	1 (0)
Unknown	0 (0)	6 (1)
Preoperative intravesical therapy	417 (30)	136 (24)
Pathologic tumor classification		
pT0	9 (1)	56 (10)
pTa	14 (1)	26 (5)
pTis	111 (8)	55 (10)
pT1	303 (22)	109 (19)
pT2	519 (37)	126 (22)
pT3	312 (22)	160 (28)
pT4	119 (9)	40 (7)
Lymph node status		
pNx	169 (12)	177 (31)
pN0, 1–10 lymph nodes	430 (31)	121 (21)
pN0, ≥11 lymph nodes	621 (45)	190 (33)
pN1/pN2	165 (12)	84 (15)
Synchronous carcinoma in situ	309 (22)	148 (26)
Multifocality	709 (51)	328 (57)
Urethral, ductal, or stromal prostatic involvement	133 (10)	75 (13)
Margin status		
Positive radical surgical margin	15 (1)	10 (2)
Positive urethral margin	37 (3)	17 (3)
Positive ureteral margin	30 (2)	23 (4)
Neoadjuvant chemotherapy	28 (2)	60 (11)
Adjuvant chemotherapy	72 (5)	139 (24)

Data presented are number of patients (%).

### Assessed outcomes

Primary end point was the site-specific recurrence after radical cystectomy. Disease recurrence to the abdomen/pelvis included local recurrence in the pelvis and distant metastasis within the abdomen. Disease recurrence to the thoracic region was defined as distant metastatic to the lungs, heart, and thoracic lymph nodes, including the mediastinal and paratracheal lymph nodes. The first recurrence to each location after cystectomy was analyzes [8].

### Development of site-specific recurrence models

Abdomen/pelvis model provides a risk estimate of abdomen/pelvis recurrence based on four histopathological variables: primary tumor stage, regional lymph node and extent of lymph node dissection, multifocality, and prostatic invasion. Thoracic region model requires three histopathological variables: primary tumor stage, regional lymph node and extent of lymph node dissection, and multifocality. Upper urinary tract model is developed based on four clinicopathological variables: primary tumor stage, multifocality, positive ureteral margin, and gross hematuria. Bone model included five clinopathological variables: primary tumor stage, regional lymph node and extent of lymph node dissection, positive urethral margin, occupational radiation exposure, and body mass index. Scoring algorithms to predict the likelihood of disease recurrence in the abdomen/pelvis, thoracic region, upper urinary tract, and bone were applied. The scores assigned each predictor are shown in Table 2.

**Table 2:** Site-specific risk stratification.

	Risk score	Risk stratification
Abdomen/pelvis	pT3 (3), pT4 (4), pNx (2), pN0 and 1–10 LN (1), pN+ (2), multifocality (2), prostatic invasion (1)	0/1–2/3/4–5/6+
Thoracic region	pT3 (4), pT4 (5), pNx (3), pN0 and 1–10 LN (2), pN+ (4), multifocality (2)	0–3/4/5–7/8–9/10+
Upper tract	pT4 (3), multifocality (2), positive ureteral margin (5), gross hematuria (−2)	−2–0/1–2/3+
Bone	pT3 (4), pT4 (4), pN+ (2), positive urethral margin (3), occupational radiation exposure (4), BMI >30 (−2)	−2/0/1–3/4–5/6+

### Statistical analysis

The predicted risk of site-specific recurrence was compared with the actual site-specific recurrence in the current study population at 3 years. We quantified the discrimination ability of each model by calculating the c-index, which is identical to the nonparametric area under the receiver operating characteristics curve [10]. A c-index of 1 indicates perfect concordance, whereas a c-index of 0.5 indicates a result equal to chance. The 95% confidence interval [CI] for each c-index was determined using a 1000-replicate bootstrap procedure. The performance of each model was also evaluated by drawing a time-dependent receiver operating characteristics curve and calculating the integrated area under the curve [11]. We plotted the Kaplan-Meier curves for freedom from site-specific recurrence, stratified by each model prediction. We conducted a decision curve analysis that was proposed by Vickers et al. [12] to assess the clinical usefulness of each model by quantifying the net benefits when different threshold probabilities were considered. Although the primary endpoint of this analysis was site-specific recurrence, overall survival also was evaluated as the secondary endpoints to better characterize the behavior of each model.

All test were two-sided and p values <0.05 were considered statically significant. Statistical analysis was performed using SPSS v.18.0 (SPSS, Chicago, IL, USA) and R, version 2.13.2 (R Foundation for Statistical Computing, Vienna, Austria).

## Results

The median follow-up in the validation cohort for site-specific recurrence model was 42.3 months (interquartile range: 23.0–89.3 months). During the follow-up period, there were 165 patients (28.8%), 85 (14.9%), 31 (5.4%), and 78 (13.6%) who recurred in abdomen/pelvis, thoracic region, upper urinary tract, and bone, respectively. The 3-, 5-, and 8-year recurrence-free survival rates in abdomen/pelvis were 74.1%, 70.7%, and 67.3%, respectively. The 3-, 5-, and 8-year recurrence-free survival rates in thoracic region were 86.4%, 82.5%, and 80.8%, respectively. The 3-, 5-, and 8-year upper urinary tract recurrence-free survival rates were 95.8%, 92.2%, and 91.3%, respectively. The 3-, 5-, and 8-year bone recurrence-free survival rates were 87.8%, 83.9%, and 82.3%, respectively. Overall survival rates for the same time points were 70.8%, 61.2%, and 57.2%, respectively.

Discrimination estimates of each model are shown in Table 3. The c-indices of abdomen/pelvis, thoracic region, upper urinary tract, and bone models 3 years after radical cystectomy were 0.69 (95% CI, 0.65–0.73), 0.69 (95% CI, 0.64–0.75), 0.61 (95% CI, 0.52–0.69), and 0.65 (95% CI, 0.59–0.71), respectively. The c-indices of each model for 5-year overall survival were also <70%. In particular, the c-index of the upper urinary tract model was 0.56.

**Table 3:** Discrimination estimates (c-indices) of risk prediction models for site-specific recurrence.

	Discrimination (95% confidence interval)	
	3-year site-specific recurrence	5-year overall suvival
Abdomen/pelvis	0.690 (0.650–0.730)	0.698 (0.666–0.730)
Thoracic region	0.692 (0.636–0.748)	0.698 (0.665–0.731)
Upper tract	0.605 (0.522–0.689)	0.556 (0.518–0.593)
Bone	0.650 (0.589–0.711)	0.660 (0.626–0.694)

To determine the accuracy of the models over the course of a follow-up period, we completed a concordance summary (integrated area under the curve). For time to recurrence for patients, integrated area under the curve values of all models were less than 70%. In particular, those of the upper urinary tract model for 3-year site-specific recurrence and 5-year overall survival were 0.61 and 0.57, respectively.

**Figure 1:**
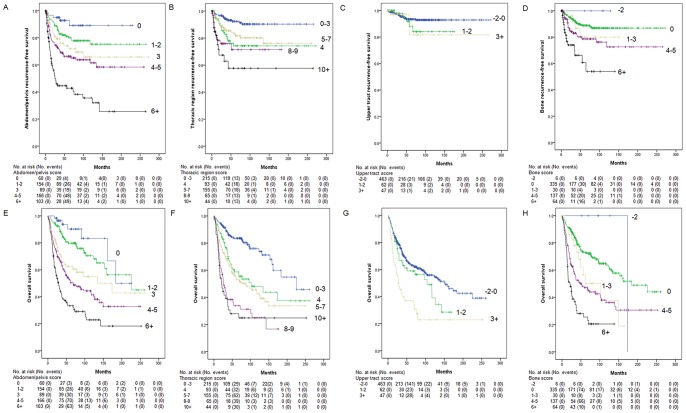
Kaplan-Meier plots. Three-year site-specific recurrence-free survival of abdomen/pelvis model (A), thoracic region model (B), upper urinary tract model (C), and bone model (D). Five-year overall survival of abdomen/pelvis model (E), thoracic region model (F), upper urinary tract model (G), and bone model (H).

**Figure 2:**
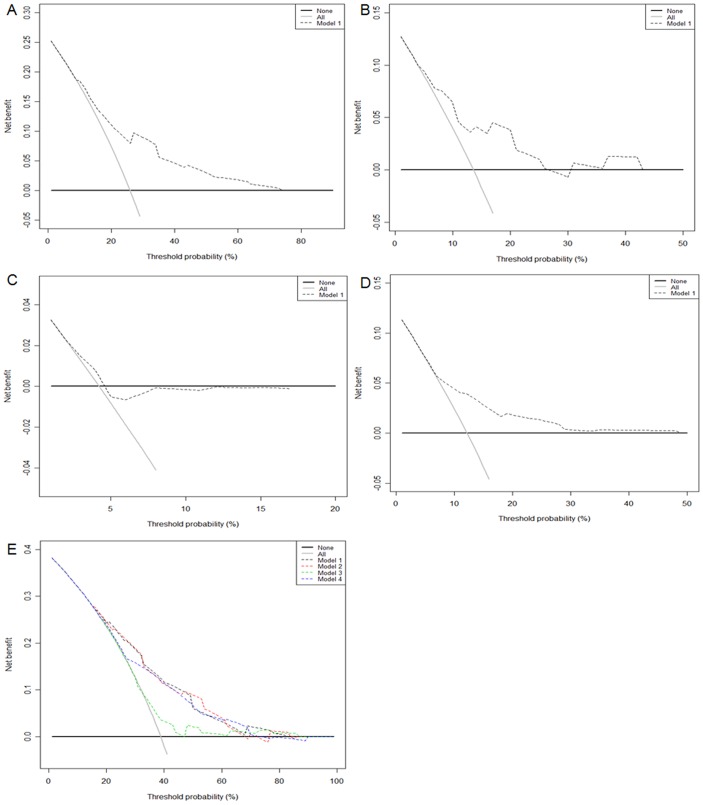
Decision curve analysis. Three-year site-specific recurrence-free survival of abdomen/pelvis model (A), thoracic region model (B), upper urinary tract model (C), and bone model (D). Five-year overall survival (E); model 1– abdomen/pelvis mode; model 2– thoracic region model; model 3– upper urinary tract model; and model 4– bone model. In decision curve analysis, the y-axis measures net benefit, calculated by summing the benefits (true positives) and subtracting the harms (false positives).

In Kaplan-Meier curves for patients stratified into groups from each model, patients were clustered into three or five groups according to their model-predicted recurrence. As depicted, models discriminated well and log-rank test were all highly significant (all p<0.001), except upper urinary tract model (p = 0.366) (Fig. 1C). Figure 2 presents the results of the decision curve analysis of site-specific recurrence at 3 years (2A-2D) and overall survival at 5 years (2E) for each model. Decision curve analysis revealed that the use of prediction models for abdomen/pelvis, thoracic region, and bone recurrence was associated with net benefit gains relative to the treat-all strategy (2A, 2B, and 2D), but not the model for upper urinary tract recurrence (2C). Also, the upper urinary tract model had a lesser net benefit for prediction of overall survival compared with other models (2E).

## Discussion

The goal of surveillance after radical cystectomy is to detect recurrence of disease as well as to identify complications. Patient performance status and the extent of visceral metastatic disease are independent prognostic factors for survival in patients with metastatic bladder cancer [13]. Therefore, surveillance to detect asymptomatic recurrent disease may improve the response to treatment by minimizing tumor burden and maximizing patient performance status at therapy. Volkmer et al. [14] failed to demonstrate a survival benefit for detecting tumor recurrence early by regular follow-up examinations. However, Giannarini et al. [5] noted that patients diagnosed with recurrence during routine follow-up had significantly improved cancer-specific and overall survival compared to patients diagnosed after symptomatic relapse. A recent study from the Mayo clinic also showed that patients who were symptomatic at recurrence had a 60% increased risk of death than those who were asymptomatic [4]. Differences in patient numbers, follow-up and exclusion of secondary urothelial tumors in the study of Volkmer et al [14] may in part account for the disparate findings.

Although modest objective response rates have been observed with cisplatin-based chemotherapy following recurrence, subsequent survival is generally highly attenuated. Previous studies suggested that aggressive surveillance following radical cystectomy is indicated since bladder cancer recurs in an unpredictable pattern [15]. On the contrary, Yafi et al. [16] proposed a stage-based protocol for surveillance of patients with bladder cancer treated with radical cystectomy that captured most recurrences while limiting over-investigation. They strongly advocated earlier surveillance in patients with extravesical and node-positive disease. However, as seen in other malignancies, recurrence and survival prediction models based on stage alone may be inadequate. An ideal surveillance schedule should rely on the natural history on the disease [17]. Post-recurrence survival course varies in patients depending on when and where their disease recurs [18]. Therefore, characterization of the patterns of recurrence following radical cystectomy is critical for patient counseling and for the development of evidence-based surveillance guidelines [19].

Because no randomized trials have compared follow-up schedules, there is no definitive data guiding follow-up post-radical cystectomy. Models for site-specific disease recurrence may be useful in generating individual patient surveillance protocols based on risk factors present at the time of cystectomy. Recently, Umbret et al. [8] examined the site-specific pattern of disease recurrence and created multifactorial scoring system to predict site-specific recurrence. In their study, site-specific disease recurrences were classified into four locations to correspond to the most commonly used diagnostic modalities in postoperative surveillance. The authors demonstrated that patients with higher scores experiences the majority of recurrences early, and few recurrences were noted after the first 3 years, suggesting that patients with higher scores should be followed closely within the first 2 to 3 years and require less vigorous follow-up thereafter [8]. Therefore, these models lend support to the use of risk-stratified follow-up and emphasize the need for earlier strict surveillance in patients with high-risk site-specific recurrence. However, these models must be externally validated in a variety of data sets, preferably from different continents in order to introduce predictive tools into the daily patient care outside of North America.

The aim of the present study was to externally validate site-specific recurrence models in Asian cohort of patients who had undergone radical cystectomy for urothelial carcinoma. First, these models were constructed with the appropriate selection of variables for inclusion in the analysis. However, discrimination accuracy of models was moderate in our cohort although all currently available predictive tools in bladder cancer are not perfectly accurate. Risk groupings for calculating clinical risk are simple and convenient, but they do pose some problems. The misconception related to this approach is that it assumes that all patients within a risk group are equal despite risk group comprise a heterogeneous group of patients. The individual heterogeneity within a risk group leads to lower predictive accuracy when applying risk classifications to a particular patient. Risk groupings present additional statistical limitations by categorizing data points. When compared to nomogram, risk grouping is an inefficient use of the data and tends to reduce the predictive accuracy of a prognostic model (spectrum bias) [20]. Second, in our validation study, the bone model did not provide sufficient accuracy. Patient factors might influence outcomes. In the development cohort, patients with a history of occupational radiation exposure had an increased risk of osseous recurrence. In the validation cohort, we could not find patients with a history of radiation exposure. Third, the c-index for predicting 3-year upper tract recurrence-free survival using upper urinary tract model was 0.61 (95% CI, 0.52–0.69). The imperfection of upper urinary tract model may have been a reflection of a different biological process of upper urinary tract recurrence since upper urinary tract recurrences represent a different biological process of recurrence, and treatment and prognosis differ greatly from those of true recurrences [6]. While most local and distant failures were observed in the first 2 years following radical cystectomy, the risk of upper urinary tract recurrence is only a problem in the long term. The finding that c-indices were relatively similar for each site except for upper urinary tract might be attributed to the relatively adequate number of actual recurrence at each site (more than 10% of the validation cohort patients, respectively) during the study follow-up period, unlike small number of patients (5.4%) showing upper urinary tract recurrence. Finally, in the second part of the study, we used site-specific recurrence models to assess their predictive accuracy for overall survival in our patient cohort. As a result, except for upper urinary tract recurrence model, the patients with higher scores were likely to show a worse recurrence-free survival and overall survival in the remaining site-specific recurrence models. Site-specific models except upper urinary tract model identified subgroups that also have significantly different overall survival, confirming the clinical importance of the stratification. Our finding suggests that upper urinary tract recurrence after radical cystectomy is not associated with patient's subsequent risk of death. However, these survival findings related to the site-specific recurrence models were not main focus of our study. The site-specific recurrence and evaluation with regard to the accuracy of site-specific recurrence models were the primary end point and main focus of our study.

The present study is limited because of its retrospective character with inherent biases. Another limitation is that this study comes from a single institution. However, despite the tertiary referral nature of our practice, most patients received postoperative follow-up at our institute. Since neoadjuvant and adjuvant chemotherapy was offered to a subset of patients (11% and 28%, respectively) in the present cohort, this may have potentially altered the recurrence patterns and influenced our surveillance strategies. Moreover, patients were not randomized to neoadjuvant or adjuvant chemotherapy, thereby introducing a selection bias. The fact that only 31 patients had upper urinary tract recurrence is a very limited number of samples for statistical analysis, and perhaps this could be the contributing factor of non-significant c-indices for this site. The different types of follow-up and sensitivity of methods to detect recurrent disease may also influence the result. Finally, 31% of patients in the validation dataset did not undergo pelvic lymph node dissection, whereas 12% in the development cohort did not. Therefore, it is possible that their application to cohorts with different clinical or pathological characteristics might result in the different performance characteristics.

## Conclusions

Our study may be meaningful in that it offers a comprehensive validation of site-specific recurrence models in a different cohort. Abdomen/pelvis, thoracic region, and bone models demonstrate moderate discrimination, adequate calibration, and meaningful net benefit gains, whereas upper urinary tract model does not seem applicable to patients from Asia because it has suboptimal accuracy.
