# Correction: Multifactorial, Site-Specific Recurrence Models after Radical Cystectomy for Urothelial Carcinoma: External Validation in a Cohort of Korean Patients

**DOI:** 10.1371/journal.pone.0107845

**Published:** 2014-09-03

**Authors:** The PLOS ONE Staff

There is an error in the first sentence of the Study cohort section of the Materials and Methods. The correct sentence is: We conducted a review of an electronic medical record of all 622 patients who underwent radical cystectomy for bladder cancer at Seoul National University Hospital from January 2001 through December 2011.
